# Supplementary material for: The interactome of intact mitochondria by cross-linking mass spectrometry provides evidence for coexisting respiratory supercomplexes
Source: Mol Cell Proteomics. 2017 Dec 8;17(2):216–32. doi: 10.1074/mcp.RA117.000470 (PMC5795388; doi:10.1074/mcp.RA117.000470)
Supplement: Supplemental Data [file supp_RA117.000470_133922_0_supp_23978_fzffwf.zip › spectra_annotation/mito_spectra_annotation/135-1-12-1-12-1.pdf]

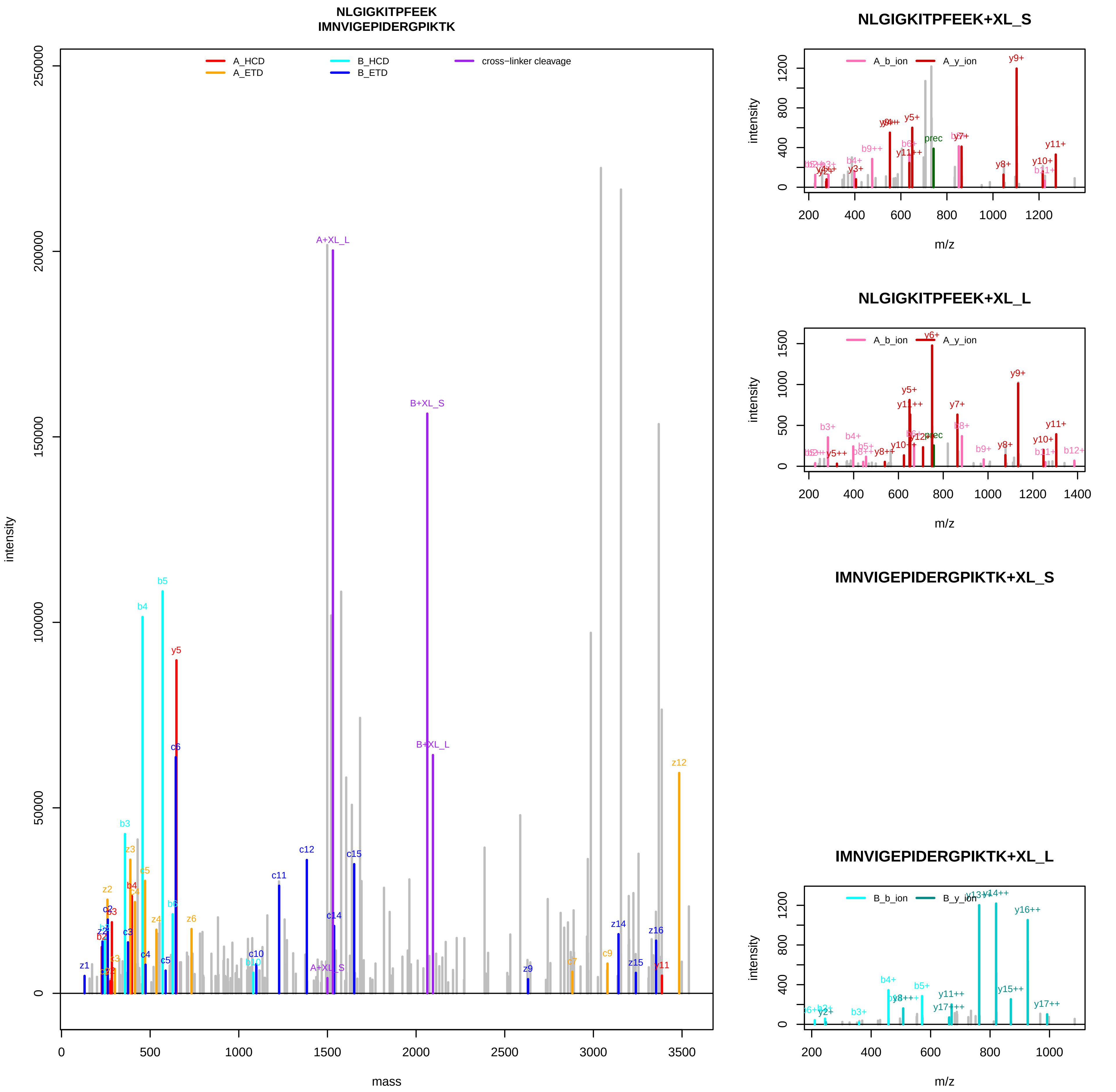
NLGIGKITPFEEK
IMNVIGEPIDERGPIKTK
A_HCD
A_ETD
B_HCD
B_ETD
cross−linker cleavage
intensity
mass
A+XL_L
B+XL_S
B+XL_L
A+XL_S
b5
b4
y5
c6
b3
z3
c5
b4
z2
c2
b3
z12
c11
c12
c15
c14
z14
z16
z15
z9
c9
y11
NLGIGKITPFEEK+XL_S
A_b_ion
A_y_ion
y9+
y5+
prec
y11+
y10+
y8+
m/z
NLGIGKITPFEEK+XL_L
y6+
y5+
y11++
y7+
y9+
y11+
y10+
b3+
b4+
b12+
IMNVIGEPIDERGPIKTK+XL_S
IMNVIGEPIDERGPIKTK+XL_L
B_b_ion
B_y_ion
y14++
y16++
y15++
y17++
b4+
b5+
b3+
y11++
y17+++
